# Ombitasvir/paritaprevir/ritonavir & dasabuvir ± ribavirin following protease inhibitors failure - a prospective multi-centre trial

**DOI:** 10.1186/s12879-020-4921-3

**Published:** 2020-04-03

**Authors:** Liat Deutsch, Inbal Houri, Ziv Ben-Ari, Amir Shlomai, Ella Veitsman, Oranit Cohen-Ezra, Assaf Issachar, Orna Mor, Yael Gozlan, Rafael Bruck, Yoram Menachem, Shira Zelber-Sagi, Helena Katchman, Oren Shibolet

**Affiliations:** 1 Department of Gastroenterology and Liver diseases, Tel Aviv Sourasky Medical Centre and Tel-Aviv University, 6 Weizmann St, 64239 Tel-Aviv, Israel; 2 Sackler Faculty of Medicine, Tel-Aviv University, Tel Aviv, Israel; 3 The liver Centre, Sheba Medical Centre, Ramat Gan, Israel; 4 The Liver Institute, Rabin Medical Centre, Beilinson Hospital, Petah-Tikva, Israel; 5 Central Virology Laboratory, Ministry of Health, Chaim Sheba Medical Centre, Ramat-Gan, Israel; 6 Department of Epidemiology and Preventive Medicine, School of Public Health, Sackler Faculty of Medicine, Tel-Aviv University, Tel-Aviv, Israel; 7 School of Public Health, Faculty of Social Welfare and Health Sciences, University of Haifa, Haifa, Israel

**Keywords:** AbbVie 3D, Direct acting anti-viral agents, HCV, Protease inhibitors failure

## Abstract

**Background:**

Hepatitis C virus (HCV) infection is a leading cause of chronic liver disease and hepatocellular carcinoma. Treatment with first generation protease inhibitors (PI) + peg-interferon (pegIFN) and ribavirin (RBV) achieved sustained virologic response (SVR) rates of 65–75% but was associated with multiple side effects. The aim of this study was to evaluate safety and efficacy of Ombitasvir/Paritaprevir/Ritonavir and Dasabuvir (3D) ± RBV in HCV genotype 1 patients that failed previous treatment with first generation PIs.

**Methods:**

An investigator-initiated, open-label, multi-centre clinical trial. HCV Genotype 1 patients who were previously null/partial responders or relapsers to telaprevir, boceprevir or simepravir+pegIFN/RBV and met eligibility criteria were included. 3D ± RBV were administrated for 12 or 24 weeks according to label. The primary outcome was antiviral response (SVR12); Secondary outcomes were patient reported outcomes, adverse events and resistance associated variants.

**Results:**

Thirty-nine patients initiated treatment according to study protocol (59% men, age 54.0 ± 8.7 years, BMI 28.7 ± 4.5 kg/m^2^). Thirty-seven (94.9%) completed the study. Thirty-five patients had genotype 1b (9 cirrhotics) and 4 had genotype 1a (2 cirrhotics). Intention-to-treat SVR12 was 92.3% and per-protocol SVR12 was 97.3%. The rate of advanced fibrosis (FibroScan® score F3–4) declined from 46.2 to 25.7% (*P* = 0.045). Abnormal ALT levels declined from 84.6 to 8.6% (*P* < 0.001). Seven patients (17.9%) experienced serious adverse events (3 Psychiatric admissions, 1 pneumonia, 1 ankle fracture, 2 palpitations), and 12 patients (30.8%) experienced self-reported adverse events, mostly weakness.

**Conclusion:**

3D ± RBV is safe and effective in achieving SVR among patients with HCV genotype 1 who failed previous first-generation PI treatment.

**Trial registration:**

NCT02646111 (submitted to ClinicalTrials.gov, December 28, 2015).

## Background

Hepatitis C viral (HCV) infection is a leading cause of liver cirrhosis, hepatocellular carcinoma (HCC) and liver transplantation [1]. Viral eradication is associated with a reduction in all-cause mortality and improvement in liver histology [2, 3].

In 2011, two first-generation direct-acting antivirals (DAAs) targeting the HCV NS3/4A serine protease (protease inhibitors–PI) – telaprevir and boceprevir – were licensed for use in patients with HCV genotype 1 (GT1) infection, and later on simeprevir, the first of the second-generation DAAs, was approved. The early-PIs required co-administration of pegylated interferon (pegIFN) and ribavirin (RBV). These triple therapy regimens achieved markedly improved sustained virologic response (SVR) rates of approximately 65–75% [4, 5] as compared to the older pegIFN+RBV regimen. However, in addition to the various contraindications to pegIFN+RBV and their known side effects, these PIs had numerous and often severe side effects, necessitating discontinuation of therapy in as many as 25% of patients.

AbbVie’s first generation all oral regimen for HCV GT1 infection included 3 DAAs with distinct mechanisms of action and non-overlapping resistance patterns to target HCV proteins essential for viral replication. Paritaprevir is a non-structural protein 3/non-structural protein 4A (NS3/NS4A) PI co-administered with the pharmacokinetic booster, ritonavir (ABT-450/r); ombitasvir (ABT-267) is a NS5A inhibitor, and dasabuvir (ABT-333) is a NS5B non-nucleoside polymerase inhibitor. In the TURQUOISE-II study, AbbVie’s 3D was administered with RBV to naïve and previously treated (pegIFN/RBV) patients with HCV GT1 infection and compensated cirrhosis, for a period of 12 or 24 weeks. SVR rates were 92 and 96%, respectively [6]. The TURQUOISE-III study showed that patients with GT1b infection achieved 100% SVR without RBV [7]. This treatment regimen reduced side effects and enabled expanding patient’s treatment eligibility. However, it was not evaluated in patients with previous first-generation PI failure. Therefore, the aim of this study was to evaluate the efficacy and safety of ombitasvir/paritaprevir/ritonavir and dasabuvir±RBV in patients with HCV GT1, who have previously failed treatment with first generation PI and pegIFN+RBV.

## Methods

### Patient selection

This was a multi-centre prospective open label trial, that included HCV GT1-infected patients who had documented first generation PI viral failure at least 12 months before enrolment (null/partial responders or relapsers). Past PI regimens included telaprevir, boceprevir or simeprevir with pegIFN/RBV. We included only virologic failures and not discontinuation due to adverse events. All patients provided written informed consent. Patients were excluded if they were co-infected with HIV or HBV, had evidence of creatinine clearance < 30 mL/min, HCC, a history of non-HCV associated chronic liver disease, a severe concurrent disease or a history of drug or alcohol abuse within 6 months prior to enrolment (confirmed by a urine drug screen at the screening visit and a positive result on the alcohol consumption questioner).

### Trial procedure

Patients were treated according to label recommendations. Enrolment was to one of four study groups according to HCV genotype (1a or 1b) and presence of cirrhosis (FibroScan® transient elastography score of F4 at baseline, e.g. > 12.5 kPa). Patients with GT1b (with/without cirrhosis) and GT1a non-cirrhotics were treated for 12 weeks. Cirrhotic GT1a subjects were treated for 24 weeks.

All patients received Ombitasvir/Paritaprevir/Ritonavir and Dasabuvir with weight based RBV except for GT1b non-cirrhotics. Scheduled visits took place in 0, 4, 12 and 24 weeks for all 4 groups. The GT1a cirrhotics had an additional visit on week 36. The visit scheduled 12 weeks after the end of treatment (EOT) was defined as “end of study” visit (EOS). Phone-call assessments for adverse events and evaluation of compliance took place at weeks 8, 16 and 20 (and also 28 and 32 for 1a cirrhotics). All visits included: physician assessment and blood tests. Baseline and EOS visits also included FibroScan® evaluation. Subjects were assessed for antiviral response, clinical outcomes, patient reported adverse events and presence and emergence of resistance associated variants.

### Primary endpoint

The percentage of patients achieving SVR12 (single last HCV RNA < 12 IU/mL 12 weeks after the last dose of medications).

### Secondary endpoints

1) The percentage of patients with viral breakthrough, defined as at least one documented HCV RNA < 12 IU/mL followed by HCV RNA ≥12 IU/mL during treatment. 2) Alterations in FibroScan® scores and ALT blood levels between baseline and EOS as a surrogate marker to liver damage. 3) Patient Reported Outcomes at baseline and EOS: A) Self-reported general health perception was estimated with a highly validated question commonly used to assess general health status [8–10]: “what is your general health status?” and the answers were: 1- “excellent” 2- “very good”, 3- “good” 4- “fine” 5- “poor”. B) WPAI Hep C v2.0 – a scoring manual for work productivity and activity impairment assessment. There are two scores, presented as percentage of impairment from 0 to 100. Higher percentages indicate greater impairment: percentage of overall work impairment due to HCV (percentage of Total Work Productivity Impairment, %TWP) and percentage of general (non-work) activity impairment due to HCV (percentage of Total Activity Impairment, %TAI). 4) Adverse events were documented in all the clinical visits and phone-call assessments. Serious adverse event (SAE) was defined as previously established by the FDA [11].

### Resistance-associated substitutions (RASs)

NS3 and NS5A RASs were examined before initiating treatment in all patients while post-treatment RAS were examined only in patients with treatment failure. All samples were stored at − 70 °C until analysis. Amplification and analysis methods were previously described [12]. Brief description can be found in Supplementary 1. All analyses were done post-hoc after study termination.

### Statistical analysis

All analyses were performed on all patients who enrolled and received at least one dose of study drugs (intention to treat analysis). Continuous variables are presented as mean ± standard deviation (SD) or median (Interquartile range [IQR]), while categorical variables are presented as number (percentage). Normal distribution was established by Kolmogorov-Smirnov test. To test differences in continuous variables between two groups the independent samples t-test or the Mann-Whitney U test were performed as appropriate. To test the differences in categorical variables the Pearson Chi-Square test was performed. Related samples were compared by McNemar test for bi-nominal distributions and paired t-test or Wilcoxon signed rank test for continuous variables. Sample size calculations were based on SVR12 rates previously published for treatment naïve and pegIFN experience patients [approximately 97% for cirrhotics and non-cirrhotics [13–16]]. Inclusion of 18 subjects in each treatment group was estimated to result in a one-sided 0.050 significance level and to have 80% power to reject the null hypothesis that the test group and the standard (historical cohort) are not equivalent (the difference in proportions, π_T_ - π_S_, is 0.100 or farther from zero in the same direction) in favour of the alternative hypothesis that the proportions in the two groups are equivalent, assuming that the expected difference in proportions is − 0.090 and the proportion in the standard group is 0.970. *P* value less than 0.05 was considered statistically significant for all analyses. All statistical analyses were performed using SPSS version 25.0 for Windows (SPSS Inc., Chicago, IL, USA).

## Results

### Study population

Between March 2016–February 2017 forty patients with HCV GT1 and past PI treatment failure were screened. One patient failed screening due to potential drug-drug interactions (clopidogrel) and 39 patients initiated treatment according to the study protocol. Thirty-seven patients completed treatment and 12 weeks of follow-up and were analysed for SVR12 (treatment was discontinued prematurely in two patients). Clinical and demographic characteristics are presented in Table 1. Out of 39 patients included, 59% were male. Average age and body mass index (BMI) were 54.0 ± 8.7 years and 28.7 ± 4.5 kg/m^2^, respectively. The average baseline viral load was 24.41*10^5^ ± 29.26*10^5^IU/ml. Advanced fibrosis (F3–4) (by FibroScan®) was present in 18 patients (46.2%) while cirrhosis (F4) was present in 11 patients (28.2%). The majority of patients were previously treated with telaprevir (53.8%), 43.6% with boceprevir and only one patient with simeprevir.

**Table 1:** Baseline clinical and demographic characteristics of the trial sample

	Entire cohort (*n* = 39)
**Male gender**^a^	23 (59.0)
**Age (years)**^b^	54.00 ± 8.7
**Body mass index (kg/m**^**2**^**)**^b^	28.67 ± 4.5
**Genotype**^a^	
**1B**	
**No cirrhosis**	26 (66.7)
**Cirrhosis**	9 (23.1)
**1A**	
**No cirrhosis**	2 (5.1)
**Cirrhosis**	2 (5.1)
**FibroScan**® **scores**^a^	
**F1–2**	21 (53.8)
**F3–4**	18 (46.2)
**Previous protease inhibitor treatment**	
**Telaprevir**	21 (53.8)
**Boceprevir**	17 (43.6)
**Simepravir**	1 (2.6)
**Haemoglobin (g/dL)**^b^	14.83 ± 1.0
**Platelets (10**^**3**^**/μL)**^b^	180.18 ± 50.9
**INR**^c^	1.00 (0.11)
**ALT (IU/L)**^c^	56 (44)
**AST (IU/L)**^c^	47 (37)
**AP (IU/L)**^c^	74 (25)
**GGT (IU/L)**^c^	61 (98)
**Bilirubin (mg/dL)**^b^	0.66 ± 0.3
**Albumin (g/dL)**^b^	4.27 ± 0.2
**Creatinine (mg/dL)**^b^	0.76 ± 0.1

^a^*n* (%); ^b^ mean ± SD; ^c^median (IQR)

### Primary outcome, treatment efficacy

**Fig. 1:**
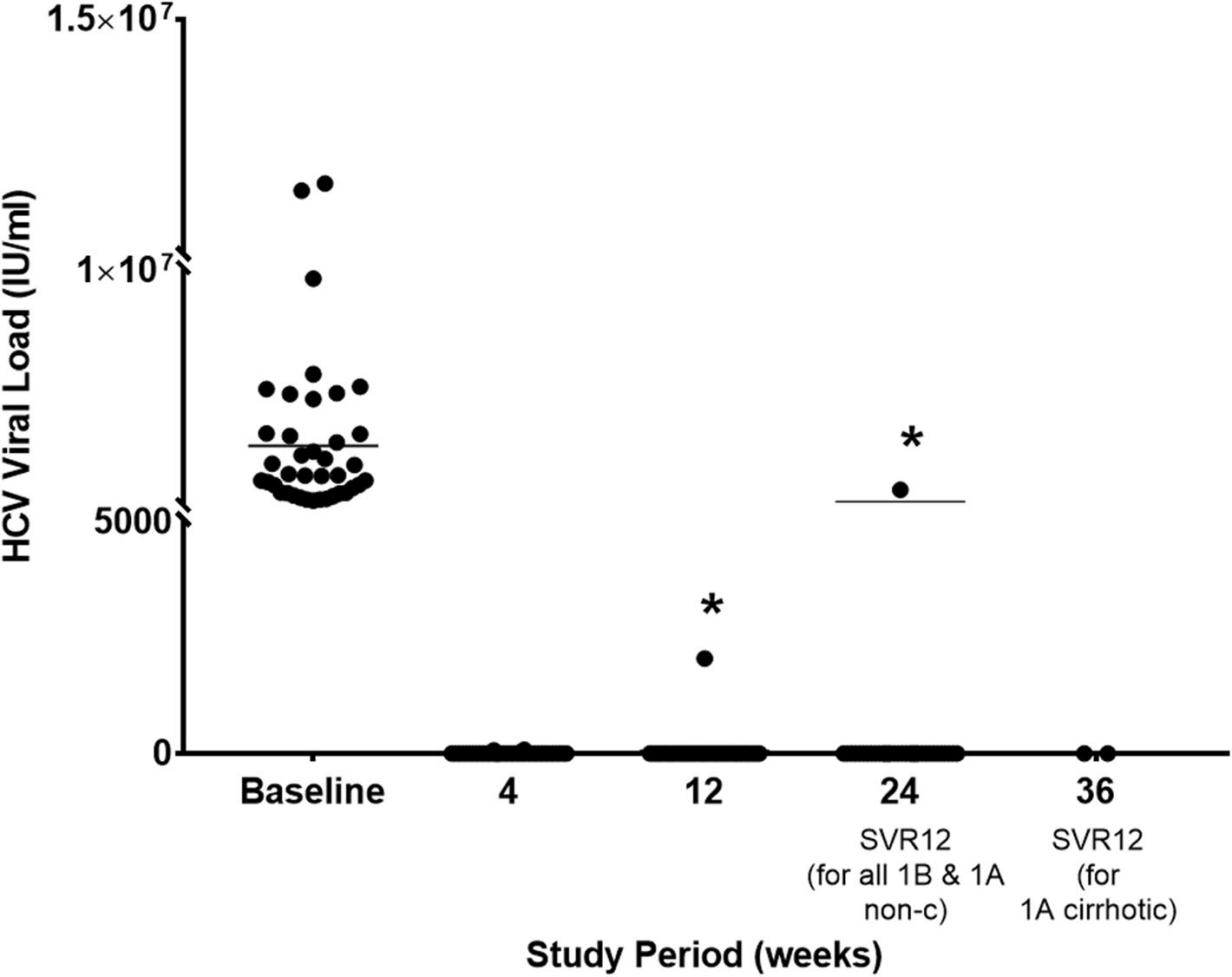
Hepatitis C Viral load. Per-protocol scatter plot of entire cohort’s patient’s viral load according to study period. * One patient had a breakthrough after 12 weeks of treatment. Non-c, non-cirrhotic

**Fig. 2:**
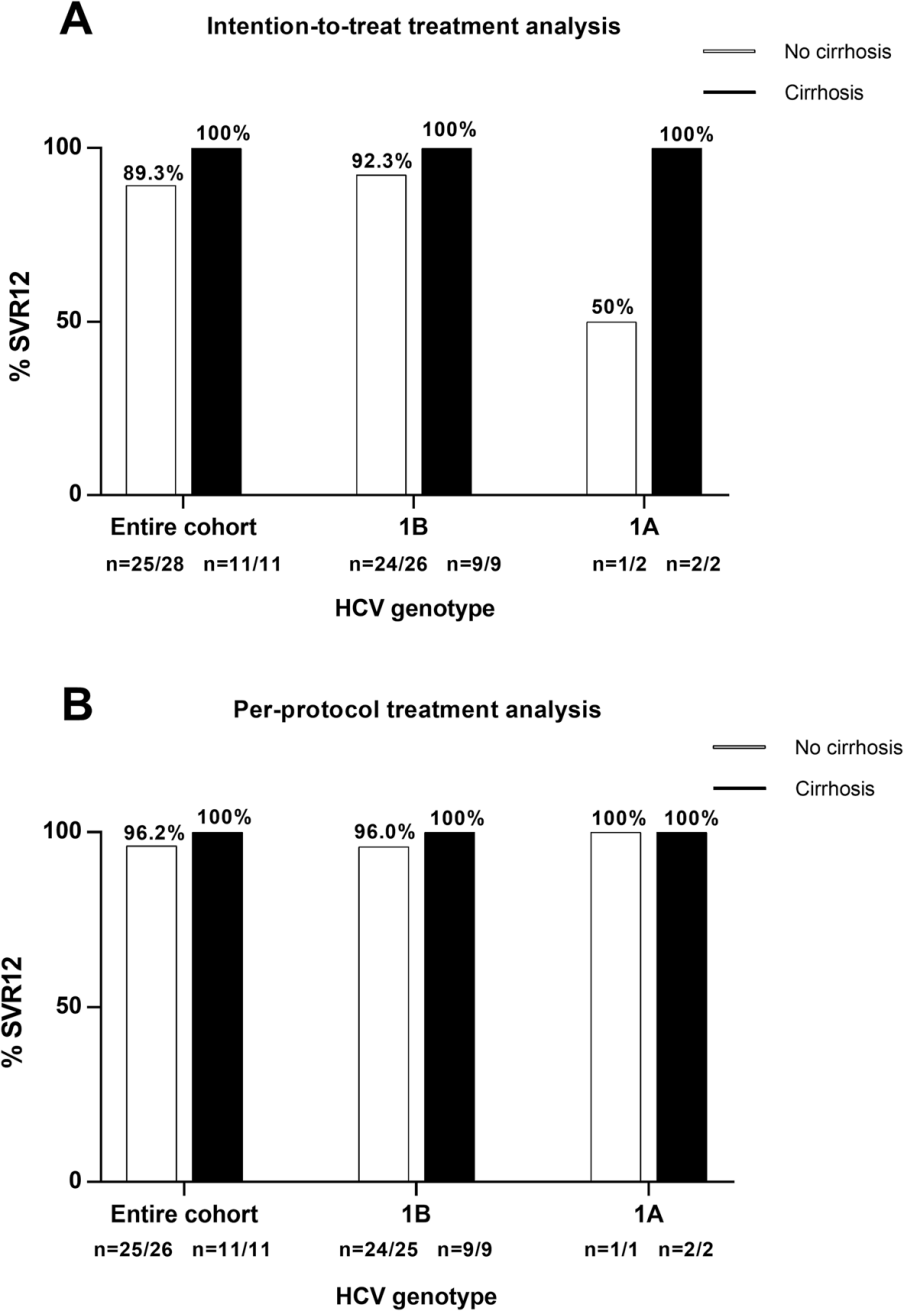
The rate of SVR12 according to HCV genotype and the presence of cirrhosis. White columns - no cirrhosis; Black columns - cirrhosis. (**a**) Intention-to treat analysis; (**b**) Per-protocol analysis

There were 4 parallel groups of treatment according to the patient’s genotype and the presence/absence of cirrhosis. Four patients had GT1a (2 cirrhotics) and 35 patients had GT1b (9 cirrhotics) (Table 1). Virologic response was observed among 36/37 patients who completed the treatment (97.3%) while one patient (2.7%, G1b- non-cirrhotic, serial no. 28) had a viral breakthrough after 12 weeks of treatment [2055 IU/ml after 12 weeks (EOT) and 554,847 IU/ml at 24 weeks, (EOS)] (Fig. 1). There was no virologic relapse among patients that completed the study, thus the per-protocol SVR12 rate was 97.3% and comparable to 97% SVR12 described in historical cohorts (*P* = 1.000, Chi-square test). There were two early treatment terminations both did not achieve SVR12. Thus, according to the intention-to-treat protocol, the SVR12 rate was 36/39 (92.3%) and comparable to the historical cohort SVR12 (92.3% vs. 97%, *P* = 0.349, Chi-square test). Intention-to-treat and per-protocol SVR12 according to treatment groups are shown in Fig. 2.

### Secondary outcomes, clinical and laboratory

**Fig. 3:**
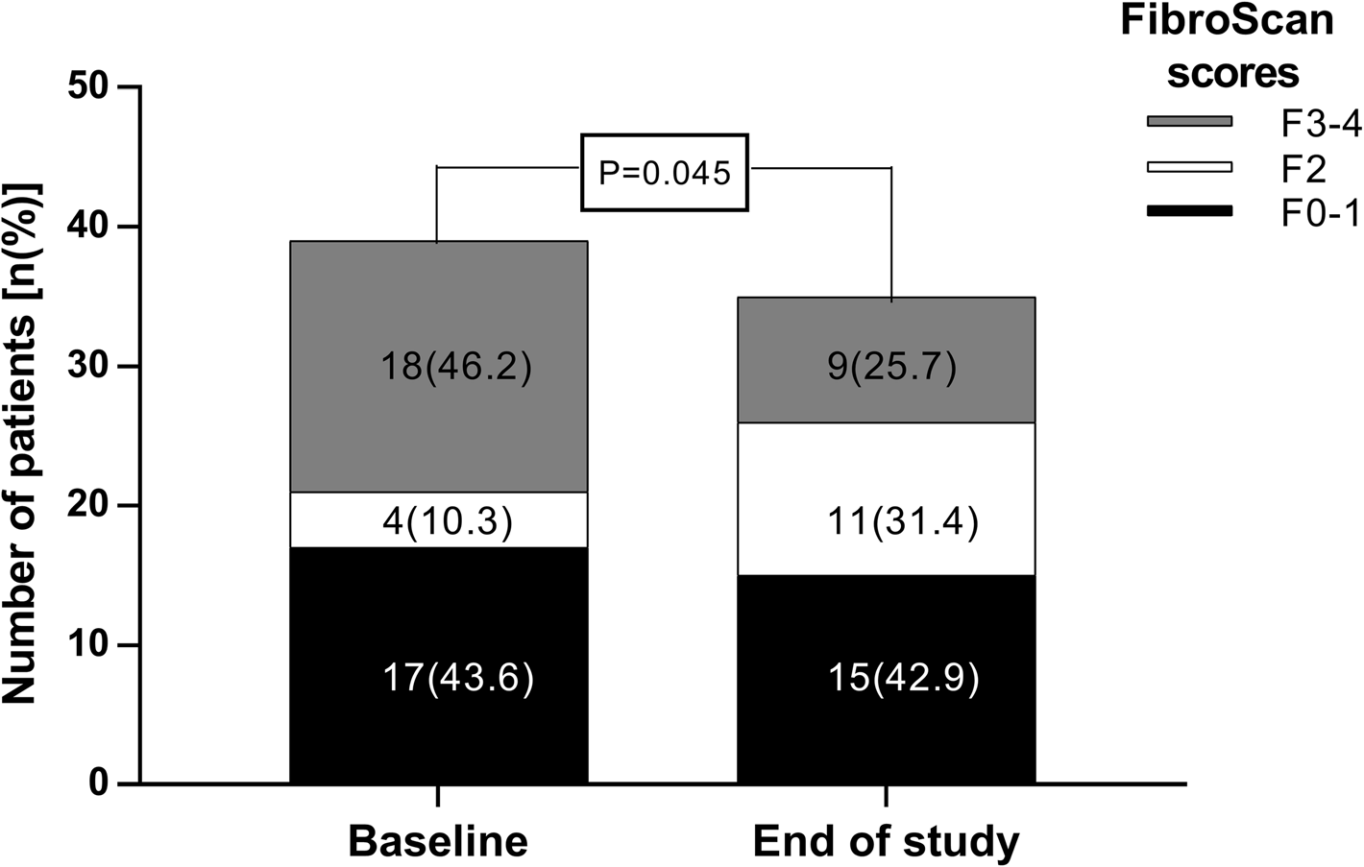
FibroScan® scores before treatment and at the end of the study. FibroScan® scores were divided to three categories: black- F0–1 - absent or mild fibrosis (< 7 kPa); white – F2 - moderate fibrosis (7 < and < 9.5 kPa); grey – F3–4 - severe fibrosis/cirrhosis (> 9.5 kPa). Baseline scores disparity was compared to end of study scores disparity (12 weeks after last treatment) (*p* = 0.045, Pearson Chi-Square test). Data is presented as number (%)

Baseline median FibroScan® score was 9.3 kPa (min-max 3.7–34.0 kPa). By EOS there was a statistically significant decrease to 7.8 kPa (min-max 3.7–34.8 kPa) (*P* = 0.007). The prevalence of advanced fibrosis (F3-F4) markedly declined from baseline 46.2% to EOS 25.7%, (*P* = 0.016) (Fig. 3). Seven patients (43.8%) had advanced fibrosis at baseline and markedly improved during treatment and follow-up (in 4 patients with F3 and 3 patients with F4 the score declined to F2). Furthermore, within the advanced fibrosis category, 2 patients with F4 at baseline improved to F3 at EOS.

**Fig. 4:**
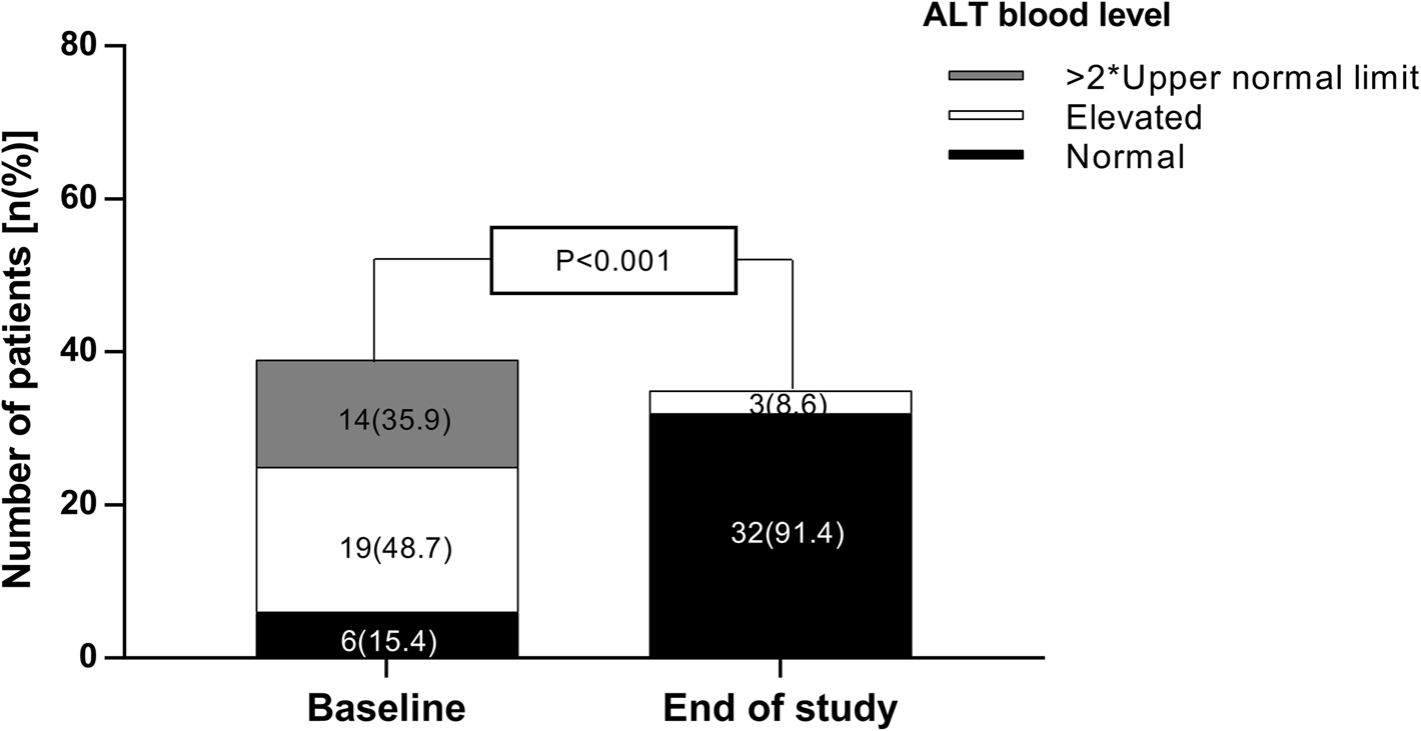
Alanine transferase (ALT) before treatment and at the end of the study. ALT blood level was divided to three categories: black-within normal range-(ALT < 35 U/L); white – elevated but less than twice the upper normal limit (35 ≤ ALT< 70 U/L); grey – more than twice the upper normal limit (ALT≥70 U/L). baseline dispersion was compared to end of study dispersion (12 weeks after last treatment) (*p* < 0.001, Pearson Chi-Square test). Data is presented as number (%)

Baseline ALT levels were abnormal in 84.6% of patients (higher than twofold the upper limit of normal (ULN) in 35.9%) while at the EOS, ALT levels were within normal range in 91.4% of the patients and none exceeded 1.5-fold of the ULN (Fig. 4).

### Patient reported outcomes

The prevalence of a better health perception (answers 1 + 2) as opposed to all other answers was comparable (39.5% vs. 41.2%, respectively, *P* = 1.000). As a continuous variable, the mean scores at baseline and at EOS were comparable as well (2.76 ± 1.0 vs. 2.64 ± 1.03, respectively, *P* = 0.513).

According to the Work Productivity and Activity Impairment Questionnaire, the prevalence of patients with a steady job at baseline and EOS was similar (74.4% vs. 71.4%, respectively, *P* = 0.625). No overall work impairment due to HCV (0% impairment) was reported by 69% of patients at baseline and 80.8% of patients at the EOS. Significant overall work impairment (≥50% impairment) was reported by 6.9% of patients at baseline and 11.5% at EOS. No impairment to general activity (0% impairment) was reported by 59.5% of patients at baseline and 64.7% at EOS. Significant impairment to general activity (≥50% impairment) was stated by 18.9% of patients at baseline and 23.5% of patients at EOS.

### Adverse events

Out of 39 patients, two patients had an early drop-out – one after a few days because of palpitations, leg oedema and general weakness and another after 6 weeks because of an acute psychotic episode and admission to a psychiatric ward. Weight-based RBV was administrated to 12/39 (30.8%) patients (1000 mg/d for 2 patients and 1200 mg/d for 10 patients). In 4 patients RBV dose had to be reduced due to side effects but none required discontinuation of the drug. The total number of any adverse events was 25 events, and 7/25 (28%) were serious adverse events (Table 2). The number of patients experiencing at least one adverse event was higher among patients treated with RBV compared to those without RBV [8 (66.7%) vs. 7(25.9%), respectively, *P* = 0.031].

**Table 2:** Adverse events

No. (%)	Entire cohort (*n* = 39)	GT1b without cirrhosis (no RBV) (*n* = 27)	GT1b with cirrhosis/ GT1a (with RBV) (*n* = 12)	*P* value
Patients with at least one AE	15 (38.5%)	7 (25.9%)	8 (66.7%)	**0.031**
Total number of AEs	25	13	12	–
Patients with any serious AE	7 (17.9%)	4 (14.8%)	3 (25.0%)	0.654
Patients with any common AE	12 (30.8)	6 (22.2)	6 (50.0)	0.133
Early termination due to AE	2 (5.1%)	1 (3.7%)	1 (8.3%)	0.526
Serious AE				
Psychiatric admission	3 (7.7%)	1 (3.7%)	2 (16.7%)	0.219
Pneumonia (admission)	1 (2.6%)	0 (0.0%)	1 (8.3%)	0.308
Ankle fracture	1 (2.6%)	1 (3.7%)	0 (0.0%)	1.000
Palpitations	2 (5.1%)	2 (7.4%)	0 (0.0%)	1.000
Common AE				
Weakness	8 (20.5%)	4 (14.8%)	4 (33.3%)	0.221
Leg oedema	2 (5.1%)	1 (3.7%)	1 (8.3%)	0.526
Rash	2 (5.1%)	1 (3.7%)	1 (8.3%)	0.526
Diarrhoea	1 (2.6%)	1 (3.7%)	0 (0.0%)	1.000
Anaemia	2 (5.1%)	2 (16.7%)	0 (0.0%)	0.089

*AE* Adverse event; *GT* Genotype; *RBV* Ribavirin

### Resistance-associated substitutions (RASs)

There were 30 pre-treatment valid samples for amplification and analysis (Supplementary 1). NS3 RASs were detected in 15 patients (50%) and NS5A RASs in 9 patients (30%). Six patients (20%) had both NS3 and NS5A RASs. Fourteen patients were previously treated with boceprevir and in two patients, resistance to boceprevir was detected (174F and 55A). One patient (patient no. 28) had resistance to boceprevir but was previously treated with telaprevir (54S, 168 V). This patient had resistance to telaprevir as well (54S, 117C). None of the other 15 patients previously treated with telaprevir had evidence of resistance. Aside from patient no. 28, four more patients had baseline resistance to telaprevir without any previous exposure. As for the current treatment, two patients had pre-treatment resistance to ombitasvir, one achieved SVR12 and the other one, patient no. 28, had dual resistance to paritaprevir as well. Post-treatment analysis was performed only to patient no. 28, which was similar to the pre-treatment pattern and showed resistance to all NS3 protease inhibitors and to all NS5A inhibitors except pibrentasvir.

## Discussion

In this open-label, prospective, multi-centre clinical trial, treatment of HCV infected patients that failed previous PI treatment with a combination of ombitasvir/paritaprevir/ritonavir and dasabuvir for 12/24 weeks ± RBV, resulted in SVR12 rates of 92% of all patients, and 97% of those who completed the treatment, similar to those reported with other second-generation treatment regimens. This study demonstrates the efficacy of 3D pegIFN-free regimen for the treatment of chronic HCV GT1 infection after previous virologic failure on first generation PIs. The study also showed marked clinical improvement following SVR. ALT levels normalized in 75% of patients with baseline abnormal levels and the prevalence of advanced fibrosis was reduced by 43% at EOS.

While this study was underway, another paper was published on 3D treatment after PI failure in a real-world setting [17]. Unlike our study, this was a retrospective observational cohort including 127 HCV GT1 infected patients who had received prior treatment with either telaprevir- or boceprevir-based triple therapy at least one-year before inclusion. All were treated with 3D + RBV for 12/24 weeks. The completion rate was 81.9%, from which 99% achieved SVR24 with only one virologic failure. RAS data was analysed only for that one patient and only before treatment and found to have NS3A/4 baseline RASs. Similar to our study, 89.5% of patients normalized their ALT levels, but there was no data regarding clinical outcomes such as change in fibrosis scores or patient reported outcomes. Moreover, in our study only patients with virologic failures were included and non-cirrhotic patients with GT1b received RBV-free treatment with excellent results, thus negating the need for this additional drug in this group of patients.

In our study, only one patient had a viral breakthrough, documented at week 12. Interestingly, this patient had pre-treatment resistance to all NS3 PIs and NS5A inhibitors, including both ombitasvir and paritaprevir. Nevertheless, this is not the exception that proves the rule regarding the necessity of RAS testing. The EASL 2018 recommendations [18] support RAS testing prior to retreatment in all DAA-treatment failures as long as the decision is made through a multidisciplinary team. However, in most cases RAS tests are not available and decisions should be guided mainly by knowledge of previous treatments with very low rate of virologic treatment failure.

Despite the obvious response to treatment, self-reported health perception was unchanged. An analysis of 8 clinical trials using the 3D regimen examined health-related quality-of-life (HRQoL) data from those studies. Patients who received this treatment reported a small but statistically significant improvement in HRQoL compared to placebo-treated patients. Patients who received combination therapy with RBV reported a decline in HRQoL during treatment [19]. The lack of significant changes HRQoL in our study may be due to a relatively small cohort, in addition to the significant number of patients who received RBV. Thirty-eight percent of the patients had an adverse event (AE) during the treatment, half of them with a serious AE. The rates of AEs in patients receiving RBV were higher, as expected. Two patients (5% of the cohort) discontinued treatment early due to AEs. Among the serious adverse events, three patients had a psychiatric event requiring hospitalization, two of whom received RBV. One had a previous psychiatric illness, and the other two had no recorded psychiatric history.

Since we conducted our study, the landscape of anti-viral treatment has markedly changed. A number of treatment options are now available for patients with GT1 after failure of prior treatment with pegIFN+RBV + early PI. all these options were shown to achieve high rates of SVR. Current EASL guidelines [18] do not endorse the use of the 3D regimen as first/second line therapy for patients that have failed previous PI treatment. However, the 3D regimen is still widely used around the world, especially in Eastern Europe where GT1 is prevalent. Furthermore, although the guidelines discourage the use of RBV, the 3D regimen may still be recommended in patients infected with GT1b without cirrhosis or with compensated cirrhosis (Child-Pugh A).

As of 2018, current guidelines by the American Association for the Study of Liver Diseases (AASLD) recommend one of three regimens for patients with GT1 who were previously treated with first generation PIs: 1. Ledipasvir/sofosbuvir [20–22], 2. Sofosbuvir/velpatasvir [23, 24] or 3. Glecaprevir/pibrentasvir [25–27], with the last two also recommended for cirrhotic patients [28]. Newer guidelines by the European Association for the Study of the Liver (EASL) recommend sofosbuvir+velpatasvir+voxilaprevir as the first choice for treatment after PI failure, defined as failure on any PI with sofosbuvir (combined with pegIFN+RBV or with RBV alone) [18]. The recommendation is based on the results of the POLARIS-1 and POLARIS-4 studies, which excluded patients who had received PIs with pegIFN+RBV [29]. Thus, it seems that for this subgroup, as were those included in our study, AbbVie’s 3D is sufficient as a second-line treatment. The professional guidelines have met with some criticism because they discourage the use of effective older medications that have a lower cost and an excellent track record. This is supported by the world health organization (WHO) report [30], stating that as of November 2017, several countries with upper-middle-income status (including Brazil, China, Colombia, Mexico, Kazakhstan and Turkey) with estimated cumulative population of 14 million people living with HCV, were not included in the license agreements to produce generic DAA and as a result cannot afford these expansive medications. Moreover, the FDA has recently published a communication regarding serious liver injury occurrence with use of Glecaprevir/pibrentasvir, Elbasvir/grazoprevir and sofosbuvir+velpatasvir+voxilaprevir in some patients with advanced liver disease [31].

## Conclusions

In conclusion, our results show that Ombitasvir/Paritaprevir/Ritonavir and Dasabuvir ±weight based RBV are safe and effective in achieving SVR among patients with HCV GT1 and failure of first generation PI treatment. Although these findings might not be of pivotal importance in many countries with unlimited access to effective all-oral combinations, this regimen, with cheaper yet effective medications, can be used as an additional option for second line treatment, especially where medication cost hinders treatment options due to limited resources.

## Supplementary information

**Additional file 1: Table S1.** NS3 Resistance-Associated Substitutions (RASs) **Table S2.** NS5A Resistance-Associated Substitutions (RASs).

## Data Availability

The datasets used and/or analysed during the current study are available from the corresponding author on reasonable request.

## Abbreviations

%TAI: Percentage of Total Work Productivity Impairment
%TWP: Serious adverse event
AASLD: American Association for the Study of Liver Diseases
AE: Adverse event
BMI: Body mass index
DAAs: Direct-acting antivirals
EASL: The European Association for the Study of the Liver
EOS: End of study
EOT: End of treatment
GT1: Genotype 1
HCC: Hepatocellular carcinoma
HCV: Hepatitis C viral
HRQoL: Health-related quality-of-life
IQR: Interquartile range
NS3/NS4A: Non-structural protein 3/non-structural protein 4A
pegIFN: Pegylated interferon
PI: Protease inhibitors
RASs: Resistance-associated substitutions
RBV: Ribavirin
SAE: Percentage of Total Activity Impairment
SD: Standard deviation
SVR: Sustained virologic response
ULN: Upper limit of normal
