# Supplementary material for: Ombitasvir/paritaprevir/ritonavir & dasabuvir ± ribavirin following protease inhibitors failure - a prospective multi-centre trial
Source: BMC Infect Dis. 2020 Apr 3;20:264. doi: 10.1186/s12879-020-4921-3 (PMC7126165; doi:10.1186/s12879-020-4921-3)
Supplement: Supplementary file 1 — Additional file 1: Table S1. NS3 Resistance-Associated Substitutions (RASs) Table S2. NS5A Resistance-Associated Substitutions (RASs). [file 12879_2020_4921_MOESM1_ESM.docx]

**Supplementary Material**

*Amplification and analysis of Resistance-associated substitutions (RASs)*

NS3 and NS5A RASs’ amplification and analysis methods were previously described ^[1]^. In brief, RNA was extracted using the NucliSENS Easy MAG total RNA extraction system (Biomerieux, Marcy l'Etoile, France) and reverse transcription PCR (RT-PCR) reactions were performed with the PrimeScriptTM One Step RT-Kit v2 (Takara Bio, Mountain View, CA 94043, United States). Amplification of GT1 NS3 was performed using a nested PCR protocol and amplification of NS5A sequences was performed using a one-step RT-PCR reaction. Subtype-specific primers were prepared according to NCBI database (Genaphora Ltd., Rehovot, Israel). Alternatively, K-16-NS5ADR assay (ABL, L-1835 Luxembourg, Luxembourg), was used for amplification and sequencing of NS5A region. Direct sequencing of all PCR products was performed using an automatic sequencer (ABI PRISM 3100 genetic analyzer DNA Sequencer, Applied Biosystems, Foster City, CA, USA) and BigDye Terminator v1.1 Cycle Sequencing kit (Applied Biosystems, Foster City, CA, USA). Nucleotide sequences were assembled using the Open-gene system (Siemens) and aligned with reference sequences for both GT1a and GT1b (GeneBank accession numbers NC_004102 or M62321 for HCV genotype GT1a, and D90208.1 for genotype GT1b). Amino acid substitutions in the NS3 and NS5A GT1a and GT1b sequences were determined using geno2pheno. Clinically relevant amino acids locations considered as RASs were: 36, 54, 55, 80, 155 and 168 in NS3 and 28, 30, 31, and 93 in NS5A ^[2]^. More than a twofold change in susceptibility to administered drugs, was considered significant.

**Supplementary Table 1. NS3 Resistance-Associated Substitutions (RASs)**

| N. | Age range (years) | GT | Past-PI | NS3 RAS | ASV | BOC | GLE | GZR | PTV | SMV | TVR | VOX |
| --- | --- | --- | --- | --- | --- | --- | --- | --- | --- | --- | --- | --- |
| 1 | 41-60 | 1b | TVR |  | S | S | S | S | S | S | S | S |
| 2 | 41-60 | 1b | TVR |  | S | S | S | S | S | S | S | S |
| 4 | 41-60 | 1b | BOC |  | S | S | S | S | S | S | S | S |
| 6 | 41-60 | 1b | BOC |  | S | S | S | S | S | S | S | S |
| 7 | ≤40 | 1b | BOC | 174F | S | R | S | S | S | S | RS | S |
| 9 | 61-70 | 1b | TVR | 122T | S | S | S | S | S | SSP | S | SSP |
| 11 | 61-70 | 1b | BOC | 56F | S | S | S | RS | S | S | S | S |
| 13 | 41-60 | 1b | TVR |  | S | S | S | S | S | S | S | S |
| 15 | 61-70 | 1b | BOC | 55A,122N | S | R | S | S | S | SSP | RS | SSP |
| 16 | ≤40 | 1b | BOC |  | S | S | S | S | S | S | S | S |
| 17 | 61-70 | 1a | BOC | 174S | S | S | S | S | S | S | RS | S |
| 18 | 41-60 | 1b | BOC | 122N | S | S | S | S | S | SSP | S | SSP |
| 19 | 41-60 | 1b | TVR |  | S | S | S | S | S | S | S | S |
| 21 | 41-60 | 1b | TVR |  | S | S | S | S | S | S | S | S |
| 22 | ≤40 | 1b | BOC |  | S | S | S | S | S | S | S | S |
| 23 | 41-60 | 1a | TVR | 174N | S | SSP | S | S | S | S | SSP | S |
| 24 | 41-60 | 1b | BOC | 117Q | S | S | S | S | S | S | SSP | S |
| 25 | 61-70 | 1b | TVR |  | S | S | S | S | S | S | S | S |
| 26 | 41-60 | 1b | TVR | 56F | S | S | S | RS | S | S | S | S |
| 27 | 41-60 | 1a | TVR |  | S | S | S | S | S | S | S | S |
| 28* | 41-60 | 1b | TVR | 54S,168V | R | R | S | RS | R | R | RS | RS |
| 31 | ≤40 | 1b | BOC | 56F | S | S | S | RS | S | S | S | S |
| 32 | 41-60 | 1b | TVR |  | S | S | S | S | S | S | S | S |
| 33 | 61-70 | 1b | TVR |  | S | S | S | S | S | S | S | S |
| 34 | 41-60 | 1a | TVR |  | S | S | S | S | S | S | S | S |
| 35 | ≤40 | 1b | BOC | 56F, 170I | S | SSP | S | R | S | SSP | SSP | SSP |
| 37 | 41-60 | 1b | BOC | 117H | S | S | S | S | S | S | RS | S |
| 38 | 41-60 | 1b | BOC | 56F,117C | S | S | S | RS | S | S | SSP | S |
| 39 | 41-60 | 1b | TVR | 56F,122G | S | S | S | RS | S | SSP | S | SSP |
| 40 | 41-60 | 1b | TVR |  | S | S | S | S | S | S | S | S |

* Identical pre- and post-treatment pattern; abbreviations: GT-Genotype; PI – Protease Inhibitor, ASV- Asunaprevir, BOC- Boceprevir, GLE- Glecaprevir, GZR- Grazoprevir, PTV- Paritaprevir, SMV – Simepravir, TVR- Telaprevir, VOX- Voxilaprevir, S-susceptible, R- resistant RS- reduced susceptibility, SSP- substitution on scored position

**Supplementary Table 2. NS5A Resistance-Associated Substitutions (RASs)**

| N. | Age range (years) | Geno-type | Fibro-Scan | Past-  PI | NS5A RAS | DCV | EBR | LDV | OBV | PIB | VEL |
| --- | --- | --- | --- | --- | --- | --- | --- | --- | --- | --- | --- |
| 1 | 41-60 | 1b | F4 | TVR |  | S | S | S | S | S | S |
| 2 | 41-60 | 1b | F4 | TVR |  | S | S | S | S | S | S |
| 4 | 41-60 | 1b | F4 | BOC |  | S | S | S | S | S | S |
| 6 | 41-60 | 1b | F4 | BOC | 31M | RS | R | R | SSP | S | S |
| 7 | ≤40 | 1b | F0-1 | BOC | 92V | SSP | S | SSP | S | S | S |
| 9 | 61-70 | 1b | F4 | TVR | 31M | RS | R | R | SSP | S | S |
| 11 | 61-70 | 1b | F2 | BOC | 93H | R | R | R | R | S | R |
| 13 | 41-60 | 1b | F4 | TVR |  | S | S | S | S | S | S |
| 15 | 61-70 | 1b | F0-1 | BOC |  | S | S | S | S | S | S |
| 16 | ≤40 | 1b | F0-1 | BOC | 58S | S | S | SSP | S | S | S |
| 17 | 61-70 | 1a | F3 | BOC |  | S | S | S | S | S | S |
| 18 | 41-60 | 1b | F0-1 | BOC |  | S | S | S | S | S | S |
| 19 | 41-60 | 1b | F0-1 | TVR |  | S | S | S | S | S | S |
| 21 | 41-60 | 1b | F4 | TVR | 92T | SSP | S | SSP | S | S | S |
| 22 | ≤40 | 1b | F0-1 | BOC |  | S | S | S | S | S | S |
| 23 | 41-60 | 1a | F4 | TVR |  | S | S | S | S | S | S |
| 24 | 41-60 | 1b | F0-1 | BOC |  | S | S | S | S | S | S |
| 25 | 61-70 | 1b | F0-1 | TVR |  | S | S | S | S | S | S |
| 26 | 41-60 | 1b | F0-1 | TVR |  | S | S | S | S | S | S |
| 27 | 41-60 | 1a | F4 | TVR |  | S | S | S | S | S | S |
| 28* | 41-60 | 1b | F0-1 | TVR | 93H | R | R | R | R | S | R |
| 31 | ≤40 | 1b | F2 | BOC |  | S | S | S | S | S | S |
| 32 | 41-60 | 1b | F0-1 | TVR |  | S | S | S | S | S | S |
| 33 | 61-70 | 1b | F2 | TVR |  | S | S | S | S | S | S |
| 34 | 41-60 | 1a | F0-1 | TVR |  | S | S | S | S | S | S |
| 35 | ≤40 | 1b | F4 | BOC |  | S | S | S | S | S | S |
| 37 | 41-60 | 1b | F0-1 | BOC |  | S | S | S | S | S | S |
| 38 | 41-60 | 1b | F3 | BOC | 58S | S | S | SSP | S | S | S |
| 39 | 41-60 | 1b | F3 | TVR | 31M | RS | R | R | SSP | S | S |
| 40 | 41-60 | 1b | F0-1 | TVR |  | S | S | S | S | S | S |

* Identical pre- and post-treatment pattern; abbreviations: GT-Genotype; PI – Protease Inhibitor, DCV-Daclatasvir, EBR- Elbasvir, LDV-Ledipasvir, OBV- Ombitasvir, PIB-Pibrentasvir, VEL-Velpatasvir, S-susceptible, R- resistant RS- reduced susceptibility, SSP- substitution on scored position

**References**

1 Gozlan Y, Ben-Ari Z, Moscona R, Shirazi R, Rakovsky A, Kabat A, Veizman E, Berdichevski T, Weiss P, Cohen-Ezra O, Lurie Y, Gafanovich I, Braun M, Cohen-Naftaly M, Shlomai A, Shibolet O, Zigmond E, Zuckerman E, Carmiel-Haggai M, Nimer A, Hazzan R, Maor Y, Kitay-Cohen Y, Shemer-Avni Y, Kra-Oz Z, Schreiber L, Peleg O, Sierra S, Harrigan PR, Mendelson E, Mor O. HCV genotype-1 subtypes and resistance-associated substitutions in drug-naive and in direct-acting antiviral treatment failure patients. Antivir Ther 2017; 22(5): 431-441 [PMID: 28067632 DOI: 10.3851/IMP3123]

2 Lontok E, Harrington P, Howe A, Kieffer T, Lennerstrand J, Lenz O, McPhee F, Mo H, Parkin N, Pilot-Matias T, Miller V. Hepatitis C virus drug resistance-associated substitutions: State of the art summary. *Hepatology* 2015; **62**(5): 1623-1632 [PMID: 26095927 DOI: 10.1002/hep.27934]
